# Comparison of bone mass and quality determinants in adolescents and young adults with juvenile systemic lupus erythematosus (JSLE) and juvenile idiopathic arthritis (JIA)

**DOI:** 10.1186/1546-0096-12-S1-P21

**Published:** 2014-09-17

**Authors:** Fernanda Falcini, Loredana Cavalli, Federico Bertini, Marco Matucci Cerinic, Maurizio De Martino, Maria Luisa Brandi, Stefano Stagi

**Affiliations:** 1 Department of BioMedicine, Section of Rheumatology, Transition Clinic, University of Florence, Italy; 2 Department of Internal Medicine, Endocrinology Unit, University of Florence, Italy; 3 Health Sciences Department, Anna Meyer Children’s University Hospital, University of Florence, Florence, Italy

## Introduction

Few prospective data have been published on the comparison of bone density and quality in homogeneous groups of patients with juvenile systemic lupus erythematosus (JSLE) and juvenile idiopathic arthritis (JIA).

## Objectives

To perform a longitudinal evaluation of the prevalence and the characteristics of bone mass and quality in JSLE patients and to evaluate the differences on the bone biomechanical parameters, using DXA, pQCT, and QUS, in respect to two homogenous for age- and sex-groups of JIA patients and healthy controls.

## Methods

Forty-three JSLE patients (35 females, 8 males, median age 18.8 years, range 14.0 – 34.1 years) have been cross-sectionally studied with DXA, pQCT, and QUS scans and compared with 138 JIA patients (112 females, 26 males, median age 18.9 years, range 13.4 - 33.2 years: 89 oligoarticular, 26 poly, 8 systemic, 15 enthesitis-arthritis (ERA), and 79 healthy subjects (59 females, 20 males; median age 19.3 years, range 13.5 to 36.5 years). Of these, 39 patients (32 females and 7 males, median age 20.3 years, range 16.6 - 36.8 years) with JSLE were followed longitudinally and compared with 131 patients (108 females, 23 males median age 20.7, range 15.8 - 37.1 years) with JIA and 63 healthy subjects (48 females, 15 males; median age 21.9 years, range 15.5 to 38.3 years).

## Results

JSLE patients have a higher bone cortical density (CrtBMD) than controls, with JIA patients (p < 0.005), except the systemic subgroup (p < 0.0001), showing a lesser CrtBMD than controls and JSLE group. However, JSLE and JIA patients, except for ERA onset, have a significantly reduced bone trabecular density (TrbBMD) compared to controls (p < 0.0001), with no differences between JSLE and JIA. In addition, JIA patients show a significantly reduced muscle area (muscle CSA) compared to JSLE and controls (p < 0.001). Conversely, fat area (fat CSA) is significantly increased in both JIA and JSLE patients when compared to controls (p < 0.001), with no differences between JSLE and JIA groups. Analogous results are observed in the polar resistance to stress (SSIp).

## Conclusion

The evaluation of the main parameters that define bone density and structure in adolescents and young adults with JIA and JSLE highlights significant differences among the two groups and subgroups, and among JIA or JSLE patients and controls. These data might indicate a different pathogenesis of bone damage in the two entities, and suggest a different diagnostic and therapeutic approach to improve the peak bone mass.

## Disclosure of interest

None declared.

